# Mitigation of Silicon Contamination in Fuel Cell Gasket Materials through Silica Surface Treatment

**DOI:** 10.3390/polym16070914

**Published:** 2024-03-26

**Authors:** Yoo Lim Sim, Jaewon Lee, Su Min Oh, Dong Beom Kim, Kijong Kim, Sung-Hyeon Baeck, Sang Eun Shim, Yingjie Qian

**Affiliations:** 1 Guangzhou Institute of Energy Conversion, Chinese Academy of Sciences, NengYuan Street 2, Tianhe District, Guangzhou 510640, China; 2 Department of Chemistry and Chemical Engineering, Education and Research Center for Smart Energy and Materials, Inha University, Incheon 22212, Republic of Korea; 22192186@inha.edu (J.L.); 22222222@inha.edu (D.B.K.);

**Keywords:** silicone rubber gasket, silica surface modification, silicone elution

## Abstract

Gaskets and seals are essential components in the operation of proton exchange membrane (PEM) fuel cells and are required for keeping hydrogen and air/oxygen within their individual compartments. The durability of these gaskets and seals is necessary, as it influences not only the lifespan but also the electrochemical efficiency of the PEM fuel cell. In this study, the cause of silicon leaching from silicone gaskets under simulated fuel cell conditions was investigated. Additionally, to reduce silicon leaching, the silica surface was treated with methyltrimethoxysilane, vinyltriethoxysilane, and (3,3,3-trifluoropropyl)trimethoxysilane. Changes in the silica surface chemistry were investigated by scanning electron microscopy, energy dispersive X-ray spectroscopy, thermogravimetric analysis, elemental analysis, X-ray photoelectron spectroscopy, and Fourier transform infrared spectroscopy. Inductively coupled plasma-optical emission spectroscopy analysis revealed that surface-treated silica was highly effective in reducing silicon leaching.

## 1. Introduction

Fuel cells are devices that generate electricity through electrochemical processes using fuels, i.e., hydrogen and oxygen, and produce clean energy. Proton exchange membrane (PEM) fuel cells are the most representative fuel cells that have high efficiency and diverse applications. PEM fuel cells consist of membrane electrode assembly (MEA), end plates, bipolar plates, gas diffusion layers, current collectors, and elastomeric gaskets [1,2]. All these components must be assembled correctly, and their edges must be sealed with sealing materials like gaskets.

Gaskets are an important component that determines the durability of a PEM fuel cell. The gasket of each cell protects the reactant gases, i.e., hydrogen and air/oxygen, within their individual compartments. Elastomeric materials, especially rubber, are mainly used as the sealing material (e.g., gaskets) because they are easy to fabricate, are relatively less expensive, and have excellent sealing properties. These sealing materials are not only in contact with acidic solutions, coolants, and moist gases but also undergo mechanical stress when used as gaskets in PEM fuel cells. Therefore, it is essential to maintain the durability and stability of gasket materials to ensure the adequate sealing performance and electrochemical performance of PEM fuel cells [3,4].

Currently, synthetic rubbers, such as ethylene propylene diene monomer (EPDM) rubber and fluorine-based rubber (FKM), are used as elastomeric materials for fuel cell gaskets [5,6]. Silicone rubber, which has excellent durability and productivity, is considered an alternative to synthetic rubber for fabricating gasket materials [7]. However, silicon-based materials are prone to degradation in a fuel cell environment (80 °C and acidic conditions), raising stability concerns. Many studies on the chemical decomposition and mechanical degradation of silicone rubber exposed to accelerated durability test solutions, i.e., high-concentration PEM fuel cell solutions, suggest that the siloxane bonds of silicon-based materials decompose in the fuel cell environment, causing chemical instability [8,9,10,11,12,13,14,15]. However, no studies have been conducted to investigate whether fillers added to silicon gaskets cause silicon leaching in the fuel cell environment.

Silica is mainly used as a filler for reinforcing silicone rubber materials [16,17,18]. Silica nanoparticles are used for reinforcing fillers owing to their economic feasibility, easy availability, and structural similarity with silicone elastomers [19,20,21,22]. Numerous studies have been conducted to produce silicone rubber composites with excellent mechanical properties. Nanoparticle properties of pure silica nanoparticles that have not been surface-treated cannot be effectively improved because particles with hydrophilic surfaces agglomerate, owing to the hydroxyl (–OH) groups present on the surface. Therefore, surface treatment is mainly used to enhance the dispersion of silica nanoparticles within the silicone rubber matrix [23,24,25,26]. The silica surface can be treated using silane coupling agents to improve the mechanical properties, thermal properties, and interactions between the filler and matrix [27,28,29,30,31].

Long-term durability and stability are important factors in PEM fuel cell performance. If the gasket degrades or the filler leaches, the performance deteriorates because the reactive gases, i.e., hydrogen and oxygen, leak and mix together, affecting the durability and lifespan of the PEM fuel cell [32]. Therefore, increasing the stability of the gasket material by minimizing silicon leaching is the most important strategy for enhancing the durability and performance of PEM fuel cells. 

In this study, the effect of fillers on silicon leaching from fuel cell gaskets was investigated, specifically focusing on commercial liquid silicone rubber (LSR) materials. Additionally, to reduce silicon leaching from gaskets under PEM fuel cell conditions, the surface of silica—a filler used in silicone rubber—was modified with silane coupling agents. Methyltrimethoxysilane (MTMS), vinyltriethoxysilane (VTES), and (3,3,3-trifluoropropyl)trimethoxysilane (TFPTMS) were used in present study to reduce the leaching of filler by improving the hydrophobicity of the filler. Furthermore, the vinyl groups in VTES could participate in a curing reaction and, as a result, the leaching of filler could be effectively reduced. Modifications in silica surface chemistry were examined by scanning electron microscopy (SEM), energy dispersive X-ray spectroscopy (EDS), thermogravimetric analysis (TGA), elemental analysis (EA), X-ray photoelectron spectroscopy (XPS), and Fourier transform infrared (FT-IR) spectroscopy. Additionally, the effect of surface-treated silica on the mechanical properties of silicone composites was investigated. Compatibility of the silicone composite was confirmed by SEM. The tensile strength, elongation at break, and hardness of the silicone composite were investigated using a universal testing machine (UTM) and a durometer. Finally, inductively coupled plasma-optical emission spectroscopy (ICP-OES) analysis confirmed that the surface-treated silica was very effective in reducing silicon leaching from silicon gaskets. This study provides insights into the mitigation of silicon contamination, contributing to the performance improvement of gaskets for PEM fuel cells. This, in turn, is expected to improve the durability and performance of PEM fuel cell systems, ultimately contributing to the development of clean and sustainable energy technologies.

## 2. Materials and Methods

### 2.1. Materials

For the commercial LSR tests, component A (SL7260A) and B (SL7260B) for two-component LSR with silica and component A (PTA) and B (PTB) for two-component LSR without silica were procured from KCC Corp., Seoul, Republic of Korea. 

For the surface modification of silica nanoparticles, fumed silica was purchased from Evonic (AEROSIL 300; specific surface area: 300 m^2^/g). MTMS (98.0%) was purchased from TCI Co., Ltd., Tokyo, Japan. VTES (98.0%) and (3,3,3-trifluoropropyl)trimethoxysilane (98.0%) were purchased from JSI silicone Corp., Seongnam, Republic of Korea. Ethanol (99.5%) and ammonium hydroxide solution (28%) were purchased from Sigma-Aldrich Corp, St. Louis, MO, USA.

### 2.2. Commercial LSR Test

The materials used in this study were commercial sealing materials with elastomeric properties, i.e., silicone rubber supplied by KCC Corp., Republic of Korea. To prepare the commercial LSR sample containing silica, equal weights of components A (SL7260A) and B (SL7260B) were mixed using a two-roll mill. Then, the mixture was poured into a metal mold and cured for 10 min at 170 °C. The cured sample was repeatedly washed with deionized water and post-cured for 4 h at 200 °C to obtain the final LSR sample containing silica. The LSR sample without silica was prepared using components A (PTA) and B (PTB) in the same manner. 

**Figure 1:**
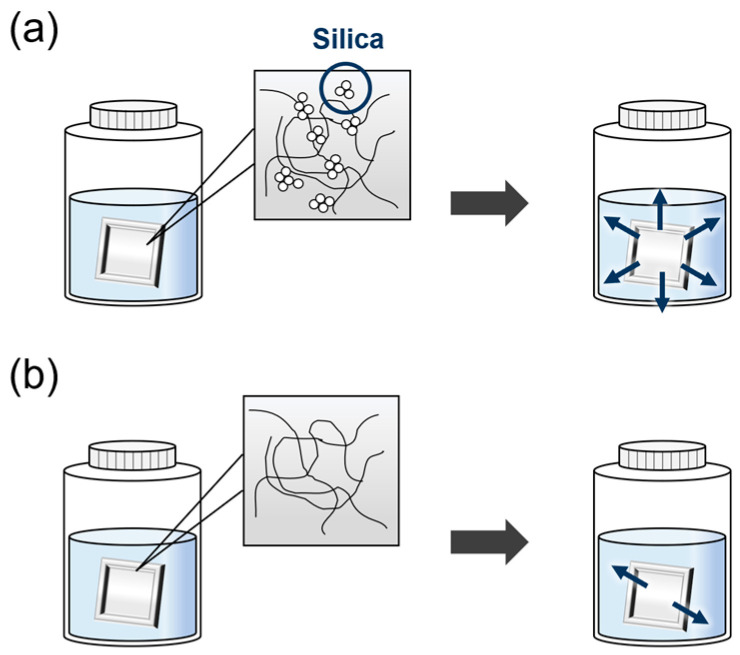
Schematic of the commercial LSR test: (**a**) LSR containing silica and (**b**) LSR without silica.

Two sheets of samples with a length and width of 30 mm and thickness of 2.0 mm were prepared. Each sample was placed in a polypropylene (PP) container and deionized water was filled into it, following which the sample was placed in an oven. The test temperature was set at 80 °C, which is close to the actual operating temperature of a PEM fuel cell. Each aged sample was removed from the test container after 168 h and the residual solution was collected for subsequent analysis. A schematic of the commercial LSR test is shown in Figure 1.

### 2.3. Surface Modification of Silica Particles

**Figure 2:**
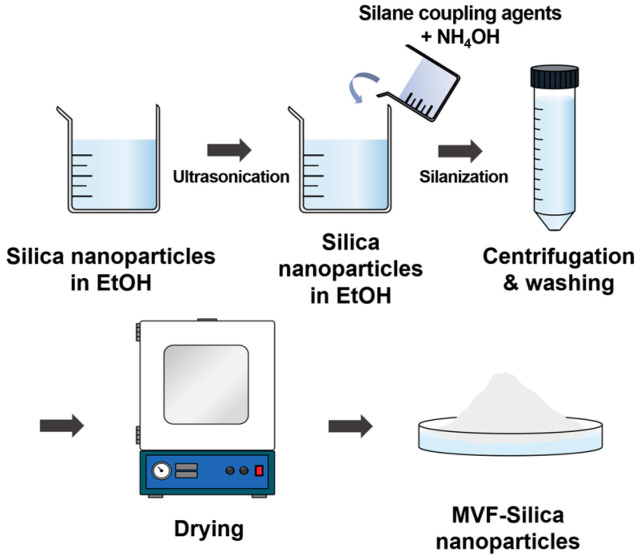
Schematic of the procedure for preparing surface-modified silica nanoparticles.

Surface treatment of fumed silica was carried out using MTMS, VTES, and TFPTMS. First, 20 g of fumed silica was dispersed into 700 mL ethanol in a 1-L four-neck flask by ultrasonicating the mixture for 30 min. After dispersion, 84 mL of ammonium hydroxide solution and each of MTMS, VTES, and TFPTMS was added to the silica suspension. Then, the mixture was heated on a heating mantle to 70 °C and stirred for 12 h. After the reaction reached completion, the mixture was centrifuged (Labogene 1248, Seoul, Republic of Korea) for 15 min at 4000 rpm and repeatedly washed with ethanol to remove impurities. This step was repeated three times. Finally, the residue was dried overnight in a vacuum oven at 60 °C to obtain the modified silica. Four types of samples were prepared using identical procedures. A schematic of this procedure is shown in Figure 2.

The surface-modified silica nanoparticles were designated as MVF-SiO_2_-1, MVF-SiO_2_-2, MVF-SiO_2_-4, and MVF-SiO_2_-16. MVF indicates that the samples had been modified with MTMS, VTES, and TFPTMS. The number in each sample name represents the amount of TFPTMS. For example, MVF-SiO_2_-1 represents silica modified with 4 g MTMS, 4 g VTES, and 1 g TFPTMS. The formulation of silane-treated silica was listed in Table 1.

### 2.4. Preparation of Silicone Rubber/Silica Filler Composites

**Figure 3:**
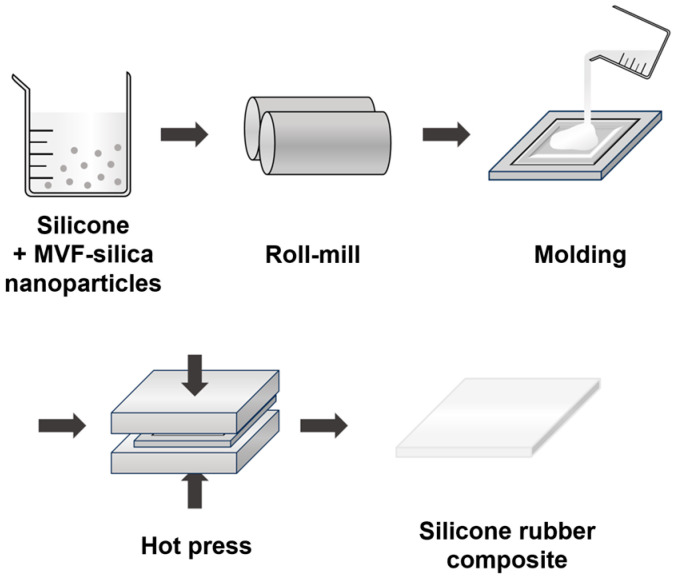
Schematic of the procedure for preparing silicone rubber/silica filler composites.

The surface-modified silica was dispersed in PTA and PTB (LSR without silica) using a two-roll mill to prepare the silicone rubber/silica filler composites. PTA and PTB were mixed at the same weight ratio, and the surface-modified silica was mixed into the composite at a ratio of 28 phr. After mixing evenly, the mixture was poured into a metal mold and cured for 10 min at 170 °C. The cured sample was repeatedly washed with deionized water and post-cured for 4 h at 200 °C to obtain the final composites. Five types of samples were prepared using identical procedures. A schematic of this procedure is shown in Figure 3.

The samples were designated as silicone rubber/SiO_2_, silicone rubber/MVF-SiO_2_-1, silicone/MVF-SiO_2_-2, silicone rubber/MVF-SiO_2_-4, and silicone rubber/MVF-SiO_2_-16.

### 2.5. Characterization 

SEM (S-4300, Hitachi, Tokyo, Japan) was used to analyze the morphologies of materials in the residual solution from the commercial silicone rubber test. It was also used to observe the morphologies of the surface-treated silica and to confirm the compatibility between silicone rubber and silica filler. EDS (S-4300, Hitachi, Japan) was used to determine the chemical composition of the surface-modified silica and the residual solution from the commercial silicone rubber test. EA (Thermo EA1112, Thermo Fisher Scientific Inc., Waltham, MA, USA) was performed for the quantitative analysis of carbon and hydrogen. FT-IR spectroscopy (VERTEX 80 V; Bruker, Billerica, MA, USA) was employed to identify the functional groups of the surface-modified silica in range of 4000–400 cm^–1^. Thirty-two scans were recorded at a resolution of 1 cm^−1^ to obtain the FT-IR spectrum. XPS (Thermo Fisher Scientific K-Alpha spectrometer, USA) was used to determine the chemical composition of the surface-treated silica using a monochromatic Al K_α_ X-ray source. TGA (TGA 4000, PerkinElmer, Waltham, MA, USA) was conducted to investigate the thermal properties of the surface-modified silica. The modified silica was heated from 30 to 880 °C under an N_2_ atmosphere at a rate of 10 °C/min. ICP-OES (Optima 7300 DV, Waltham, MA, USA) was used to detect silicon leachants.

A universal testing machine (DUT-2TC, Daekyung Engineering Co., Bucheon, Republic of Korea) was used to evaluate the tensile strength and elongation at break (ASTM D412) of the silicone rubber/silica composites. The tensile strengths were measured at a cross head speed of 200 mm/min. A durometer (TECLOCK, Tokyo, Japan) was used to evaluate the hardness (Shore A) of the silicone rubber/silica composites. The tensile strength and harness tests were conducted five times to obtain accurate values, and the average of the obtained values was calculated.

## 3. Results and Discussion

### 3.1. Contamination of Filler 

Fillers are required to enhance the mechanical properties, including tensile strength and hardness, of elastomeric materials employed for sealing purposes or for fabricating gaskets. However, filler materials like silicon dioxide might leach out from the gasket into the immersion solution under simulated PEM fuel cell conditions [3]. ICP-OES was used to detect silicon leachants from the two residual solutions collected from the commercial LSR test.

Table 2 shows the silicon concentration in the residual solution of commercial LSR materials (silicone rubber with or without silica). 

Silicon concentrations in the silicone with and without silica were 122.8 and 2.3 mg/L, respectively. The silicon contamination was likely due to leaching of the silica filler.

**Figure 4:**
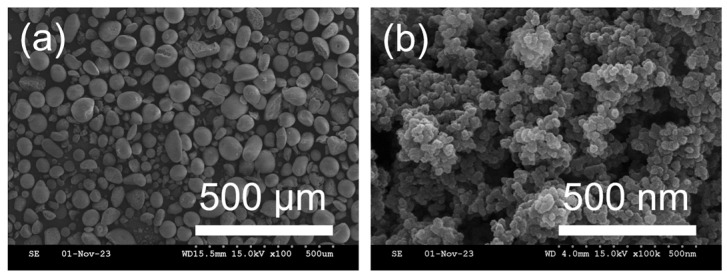
SEM microphotographs of the material in the residual solution from the commercial silicone rubber containing silica: (**a**) low magnification and (**b**) high magnification.

SEM was used to analyze the morphologies of materials in the residual solution obtained from the commercial silicone rubber test. After the residual solution was sufficiently dried in an oven, the surface of the remaining material was observed. Figure 4 shows the morphology of the material in the residual solution obtained from the commercial silicone rubber containing silica.

Figure 4a shows clustered particles, while Figure 4b shows the agglomeration of particles in the size range of tens of nanometers. These images suggest that the materials in the residual solution from the commercial silicone rubber containing silica could be silica nanoparticles.

Table 3 shows the atomic concentration of the material in the residual solution obtained from the commercial silicone rubber containing silica, according to EDS analysis. 

Silicon, oxygen, and carbon were detected in the EDS analysis of the residual solution of the commercial silicone rubber containing silica. Carbon signals probably originated from the carbon tape used to fix the materials on it. These results confirmed that the materials in the residual solution were silica nanoparticles. Consequently, it was speculated that silica nanoparticles—the filler material in commercial silicone rubber—leached out into the immersion solution under simulated PEM fuel cell conditions. 

### 3.2. Synthesis of Surface-Modified Silica

**Figure 5:**
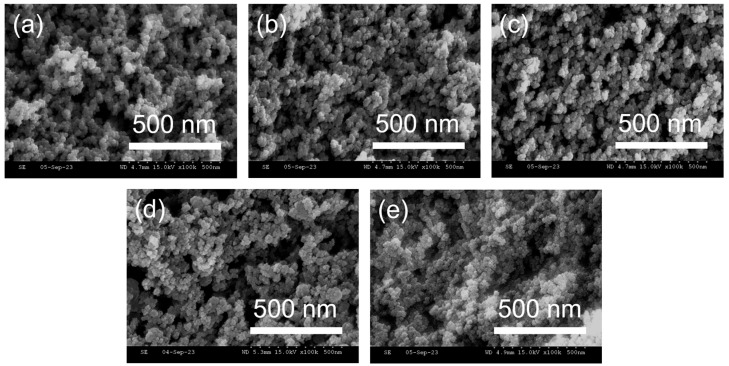
SEM micrographs of (**a**) fumed silica, (**b**) MVF-SiO_2_-1, (**c**) MVF-SiO_2_-2, (**d**) MVF-SiO_2_-4, and (**e**) MVF-SiO_2_-16.

The morphologies of the fumed silica and surface-modified silica were analyzed by SEM. Figure 5 shows SEM micrographs of the surface morphologies of silica before and after surface modification. The morphology of the fumed silica is shown in Figure 5a and the morphologies of the silica surface treated with MTMS, VTES, and TFPTMS are shown in Figure 5b–e.

A higher extent of agglomeration was observed in the surface-treated silica samples (Figure 5b–e) than in the fumed silica (Figure 5a). Additionally, the aggregation increased in the order MVF-SiO_2_-1 < (c) MVF-SiO_2_-2 < MVF-SiO_2_-4 < MVF-SiO_2_-16. Thus, the agglomeration increased with an increasing content of silane coupling agents.

Table 4 shows the quantitative EDS analysis results for silica before and after surface treatment.

The EDS analysis data show the atomic contents of silicon, oxygen, carbon, and fluorine in each sample. Fumed silica contained 34.21 at.% silicon, 57.13 at.% oxygen, and 8.66 at.% carbon. MVF-SiO_2_-1 contained 24.17 at.% silicon, 51.92 at.% oxygen, 22.71 at.% carbon, and 1.20 at.% fluorine. MVF-SiO_2_-2 contained 24.01 at.% silicon, 47.63 at.% oxygen, 26.68 at.% carbon, and 2.08 at.% fluorine. MVF-SiO_2_-4 contained 16.23 at.% silicon, 47.55 at.% oxygen, 32.23 at.% carbon, and 3.99 at.% fluorine. MVF-SiO_2_-16 contained 16.09 at.% silicon, 42.05 at.% oxygen, 33.91 at.% carbon, and 7.95 at.% fluorine. As shown in Table 4, the atomic contents of silicon, oxygen, carbon, and fluorine in each sample showed a consistent trend. The silicon content decreased from 34.21 to 16.09%, while the oxygen content decreased from 57.13 to 42.05%. However, the carbon content increased from 8.66 to 33.91% and the fluorine content increased from 0 to 7.95%. Since MTMS, VTES, and TFPTMS have carbon and fluorine in their side chains, carbon and fluorine content increased as the amount of silane used for surface treatment increased. Furthermore, as the –OH groups in the fumed silica were transformed through surface modification into –CH_3_, –CH=CH_2_, and –CH_2_CH_2_CF_3_ groups, the silicon and oxygen content decreased. These results confirm that the silica surface treatment was successful.

**Figure 6:**
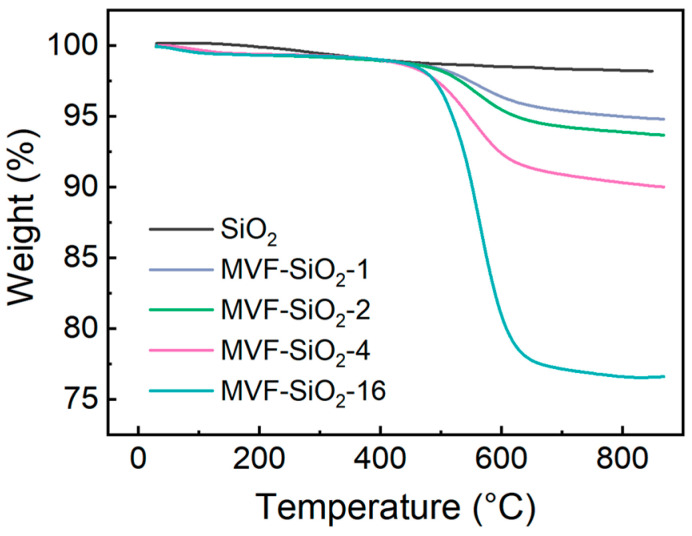
TGA thermograms of fumed silica and surface-modified silica.

TGA was employed to measure the organic content on the silica surface. Using TGA, it is possible to compare the organic content in similar types of samples. Figure 6 shows the TGA weight loss curves of silica before and after surface modification.

The weight of all samples decreased slightly as the temperature increased from room temperature to 160 °C. This could be mainly due to the evaporation of adsorbed water and the condensation of surface ≡Si–OH groups, as they thermally aggregate to release water molecules. Both processes might be promoted as the surface –OH concentration increases [33]. From 160 to 880 °C, the weight of the fumed silica decreased slightly, although there was no noticeable change. However, the weight of the surface-treated silica (i.e., MVF-SiO_2_) decreased significantly as the temperature increased from 400 °C. Furthermore, as the quantity of the silane coupling agent used in the reaction increased, the weight of the silica decreased proportionally. Thus, the higher the amount of MTMS, VTES, and TFPTMS used for silica surface treatment, the higher the number of –CH_3_, –CH=CH_2_, and –CH_2_CH_2_CF_3_ groups attached to the silica surface. As the temperature increased, these organic groups decomposed, resulting in a significant weight loss. These results confirm that the silica surface modification was successful. 

EA was employed to analyze the organic content of the silica before and after surface treatment. Table 5 shows the results of the quantitative analysis of carbon and hydrogen in silica before and after surface treatment.

As shown in Table 5, the atomic content of carbon and hydrogen in each sample showed a consistent trend. The carbon content increased from 0.20 to 16.04 wt.%, while the hydrogen content increased from 0.21 to 2.31 wt.%. Since MTMS, VTES, and TFPTMS have carbon and hydrogen in their side chains, the content of these elements increased as the amount of silane used for surface treatment increased. Thus, as the –OH groups in the fumed silica were transformed through surface modification into –CH_3_, –CH=CH_2_, and –CH_2_CH_2_CF_3_ groups, the carbon and hydrogen content increased. This result confirms the successful surface treatment of silica.

**Figure 7:**
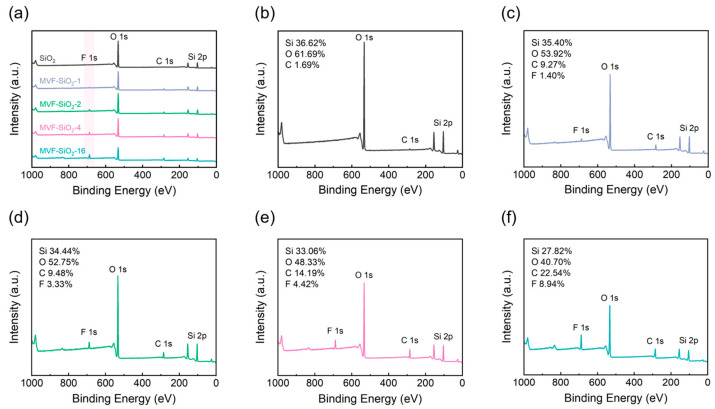
XPS analysis of silica before and after surface treatment, the survey spectrum of treated silica samples (**a**), SiO_2_ (**b**), MVF-SiO_2_-1 (**c**), MVF-SiO_2_-2 (**d**), MVF-SiO_2_-4 (**e**), and MVF-SiO_2_-16 (**f**).

XPS was used to determine the chemical composition of the silica before and after surface treatment. XPS provides a more accurate atomic content of silicon, oxygen, carbon, and fluorine than EDS. Figure 7 shows the results of the XPS analysis of the silica composition before and after modification.

The XPS spectrum of the fumed silica shows only silicon, oxygen, and carbon peaks, while that of the surface-modified silica shows silicon, oxygen, carbon, and fluorine peaks (Figure 7a). As the quantity of the silane coupling agent used in the reaction increased, silicon and oxygen peak intensities decreased, whereas carbon and fluorine peak intensities increased. Additionally, the atomic content of silicon, oxygen, carbon, and fluorine in each sample showed a consistent trend (Figure 7b–e), similar to that observed in the EDS analysis. The silicon content decreased from 36.62 to 27.82%, while the oxygen content decreased from 61.69 to 40.70%. However, the carbon content increased from 1.69 to 22.54% and the fluorine content increased from 0 to 8.94%. This trend indicated that, through silica surface treatment, the –OH groups on the surface of silica were transformed to –CH_3_, –CH=CH_2_, and –CH_2_CH_2_CF_3_ groups, resulting in a decrease in the oxygen content and an increase in the carbon and fluorine content. Thus, the XPS results further confirm the successful surface modification of silica. 

**Figure 8:**
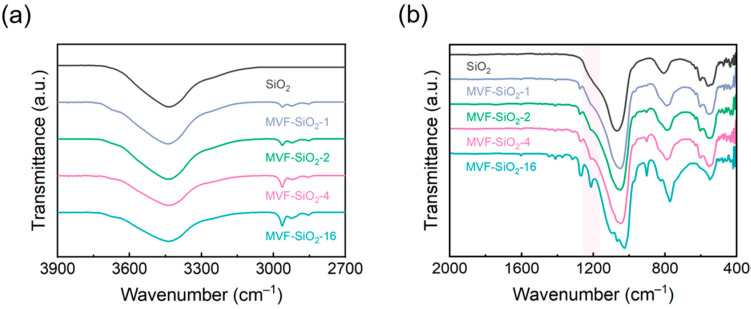
FT-IR spectra of silica before and after surface treatment in the range of (**a**) 3900–2700 cm^−1^ and (**b**) 2000–400 cm^−1^.

The reaction between the fumed silica and MTMS, VTES, and TFPTMS was monitored by FT-IR spectroscopy. Figure 8 shows the FT-IR spectra of silica before and after surface modification. The functional groups of the surface-modified silica can be identified from the FT-IR spectrum.

A new band at 2960 cm^−1^ was observed after the modification of silica (Figure 8a). This could be attributed to the C–H antisymmetric stretching of –CH_3_, indicating that –CH_3_ groups were formed on the silica surface through the reaction between silica and the silane coupling agent. Additionally, the band at 3450 cm^−1^ was attributable to an Si–OH group; this band weakened with the modification of silica. Because all samples were well dried prior to FT-IR analysis, this reduction in band intensity was mainly due to a significant reduction in the surface concentration of the –OH groups upon treatment [33]. A strong absorption band was observed from 1130 to 1000 cm^−1^ and at 800 cm^−1^ in all the spectra (Figure 8b); this was attributable to the –Si–O–Si– asymmetric stretching vibration of silica. No absorption bands corresponding to the –CF_3_ (1210 cm^−1^) and −CH_2_CH_2_− (1315 cm^−1^) groups were found in the spectrum of pure fumed silica, but were observed for MVF-SiO_2_ owing to the grafting of TFPTMS. In the spectrum of the surface-modified silica, the new peak at ~1405 cm^−1^ could be attributed to the –Si-CH=CH_2_ bond. This indicated that VTES was successfully attached to the silica surface [34]. After surface modification, new peaks corresponding to the –Si–C and C–H groups were observed at 900 and 1275 cm^−1^, respectively [35]. Furthermore, the peak at ~1616 cm^−1^ corresponded to –CH=CH_2_ absorption, which was absent in fumed silica but present in MVF-SiO_2_ owing to the grafting of VTES [36,37]. Thus, the FT-IR analysis confirmed all functional groups of the fumed silica, MTMS, VTES, and TFPTMS, and the successful surface modification of silica.

### 3.3. Compatibility of Silicone Rubber/Surface-Modified Silica Composites

**Figure 9:**
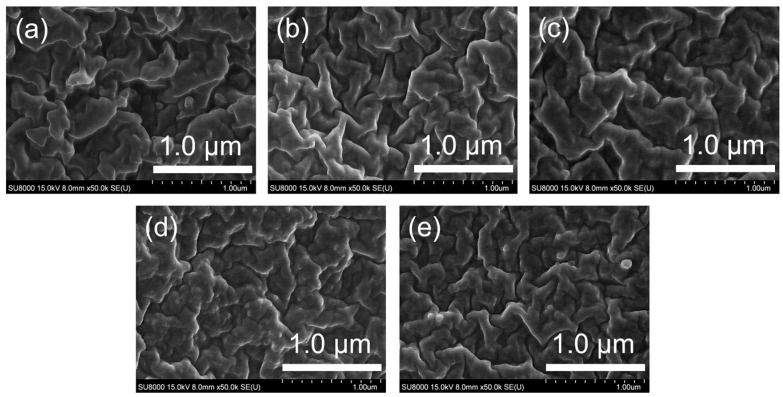
SEM micrographs of (**a**) silicone rubber/fumed silica, (**b**) silicone rubber/MVF-SiO_2_-1, (**c**) silicone rubber/MVF-SiO_2_-2, (**d**) silicone rubber/ MVF-SiO_2_-4, and (**e**) silicone rubber/MVF-SiO_2_-16.

In composite materials, compatibility is one of the crucial factors that influence material properties. Thus, the compatibility between the filler and polymer matrix is important. The compatibility between the silica filler and silicone rubber was examined by SEM. Figure 9 shows the SEM micrographs of the cross section of the silicone rubber/silica composites.

As shown in Figure 9, the cross-sectional morphologies of all samples show that the silica was well dispersed in the silicone rubber without agglomeration. Comparison of the silicone rubber/fumed composite (Figure 9a) with the silicone rubber/MVF-SiO_2_ composites (Figure 9b–e) suggested that the interaction between the silica filler and silicone rubber was excellent, even after the surface treatment of silica. However, there was a slight decrease in compatibility as the amount of TFPTMS increased (Figure 9b–e). Therefore, MVF-SiO_2_-1 and MVF-SiO_2_-2 were considered to be superior fillers compared to MVF-SiO_2_-4 and MVF-SiO_2_-16 in terms of compatibility.

### 3.4. Mechanical Properties of Silicone Rubber/Surface-Modified Silica Composites 

**Figure 10:**
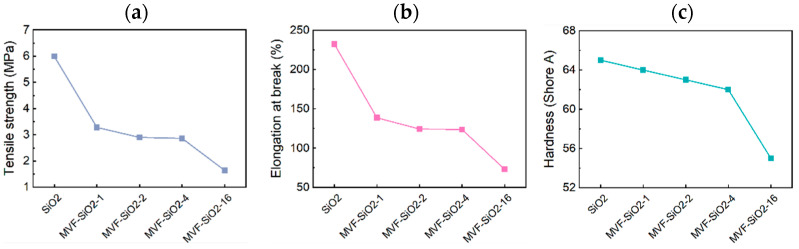
Mechanical properties of silicone rubber/silica filler composites: (**a**) tensile strength, (**b**) elongation at break, and (**c**) hardness.

The influence of the fumed silica and surface-modified silica on the mechanical properties—namely, tensile strength, elongation at break, and hardness—of the silicone rubber/silica filler composites was examined (Figure 10 and Table 6). 

To investigate how the modified silica affected the mechanical properties of the composites, all samples were prepared using the same silicone rubber and the same amount of silica filler. The reinforcing effect of silica nanoparticles on silicone rubber is significantly associated with the dispersion of fillers in the matrix [38,39]. A mechanical reinforcing effect of a composite can be expected when silica is well dispersed within the silicone rubber. As shown in Figure 6, a uniform distribution of silica particles in the silicone rubber is evident when compared to MVF-SiO_2_ particles. Therefore, the composite containing fumed silica, which was better dispersed in the silicone rubber, showed better mechanical properties than the composite containing MVF-SiO_2_. Thus, the mechanical properties degraded as the content of TFPTMS increased. Additionally, the amount of silane coupling agents used for the silica surface treatment may have influenced the mechanical properties of silicone rubber/silica filler composites. The increase in MTMS, VTES, and TFPTMS content indicated an increase in low-viscosity silane content, which can be expected to have a similar effect as that in the increase in silicone oil content. Therefore, as the amount of silane coupling agents used during the surface treatment of silica increased, the tensile strength, elongation at break, and hardness of the composites decreased [40]. 

### 3.5. Silicon Elution of Silicone Rubber/Surface-Modified Silica Composites

To compare the leaching of fumed silica and surface-modified silica from the silicone rubber composites under simulated PEM fuel cell conditions, a test was conducted using the same method as that for the commercial LSR test. Silicone rubber/silica filler composite samples were placed in a PP container filled with deionized water and were subsequently placed in an oven. The test temperature was set at 80 °C. Each aged sample was removed from the test container after 168 h, and the residual solutions were collected for subsequent analysis. The silicon concentration (Table 7) of the five residual solutions obtained from the silicone rubber/silica filler composite materials was determined by ICP-OES.

The silicon concentration in the silicone rubber containing fumed silica was 65.5 mg/L, which is lower than that in commercial silicone rubber containing silica. However, this concentration is still significantly high. For the silicone rubber containing surface-modified silica, i.e., MVF-SiO_2_, silicon leaching decreased significantly to less than 10% of that in the silicone rubber containing fumed silica. In addition, silicon leaching decreased as the content of TFPTMS increased. Thus, the TFPTMS coupling agent was effective in reducing silicon leaching. These results also suggest that silica containing highly electronegative fluorine groups endowed hydrophobicity to the composite sample and was, therefore, effective in preventing hydrolysis, which is promoted in a fuel cell environment. 

## 4. Conclusions

In conclusion, this study has demonstrated that the cause of silicon leaching in silicone gaskets used in PEM fuel cells was the contamination of silica, a filler. This study focused on reducing silicon leaching through silica surface treatment using MTMS, VTES, and TFPTMS coupling agents. The results of this study can be summarized as follows:

(1) Contamination of the filler: silicon contamination of silicone gaskets under simulated PEM fuel cell conditions occurred owing to the leaching of silica, a common filler used to improve the mechanical properties of elastomeric gaskets.
(2) Synthesis of surface-treated silica: Surface treatment of silica was successfully performed using three different silane coupling agents, MTMS, VTES, and TFPTMS. Various analytical techniques, such as SEM, EDS, TGA, EA, XPS, and FT-IR spectroscopy, were employed to examine changes in surface chemistry and, hence, confirm surface modifications.
(3) Compatibility of silicone rubber/surface-modified silica composites: The compatibility between the silicone rubber and surface-treated silica was confirmed by SEM. Results show that the fillers were well dispersed within the polymer matrix without agglomeration, confirming good compatibility. However, a slight decrease in compatibility was observed as silane content increased.
(4) Mechanical properties: The mechanical properties—namely, tensile strength, elongation at break, and hardness—of the silicone rubber composites were investigated. The mechanical properties of the composite containing fumed silica, which exhibited better dispersion, were superior to those containing MVF-SiO_2_. Moreover, the mechanical properties deteriorated as the content of the silane coupling agent increased.
(5) Silicon contamination: Silicon leaching from the silicone rubber composites was investigated by ICP-OES. Silicon leaching was significantly reduced in surface-treated silica, i.e., MVF-SiO_2_, compared to that in the composite containing fumed silica. Furthermore, the reduction in silicon leaching was more pronounced at higher contents of the silane coupling agent. These results confirm that silica surface treatment was effective in preventing the leaching of silica filler under PEM fuel cell conditions.

Overall, the surface treatment of silica with silane coupling agents is an effective approach to enhance the compatibility of silicone rubber composites and to reduce silicon leaching in PEM fuel cell environments. These findings have implications for improving the durability and reliability of gaskets and sealing materials in such applications. The use of surface-modified silica with fluorine-containing groups is expected to be particularly promising for preventing the hydrolysis of silicone gaskets in PEM fuel cell environments.

## Figures and Tables

**Table 1:** Composition of silica and each silane used for surface treatment.

	Fumed Silica (g)	MTMS (g)	VTES (g)	TFPTMS (g)
SiO_2_	20	-	-	-
MVF-SiO_2_-1	20	4	4	1
MVF-SiO_2_-2	20	4	4	2
MVF-SiO_2_-4	20	4	4	4
MVF-SiO_2_-16	20	4	4	16

**Table 2:** Silicon concentration in the residual solution collected from the commercial LSR test according to ICP-OES analysis.

	Silicon Concentration (mg/L)
Silicone containing silica	122.8 ± 3.3
Silicone without silica	2.3 ± 0.2

**Table 3:** EDS analysis of the material in the residual solution obtained from the commercial silicone rubber containing silica.

	Element (at.%)		
	Silicon	Oxygen	Carbon
Silicone with silica	32.42	58.06	9.52

**Table 4:** EDS analysis of silica before and after surface treatment.

Sample	Element (at.%)			
	Silicon	Oxygen	Carbon	Fluorine
SiO_2_	34.21	57.13	8.66	-
MVF-SiO_2_-1	24.17	51.92	22.71	1.20
MVF-SiO_2_-2	24.01	47.63	26.68	2.08
MVF-SiO_2_-4	16.23	47.55	32.23	3.99
MVF-SiO_2_-16	16.09	42.05	33.91	7.95

**Table 5:** EA analysis of silica before and after surface treatment.

Sample	Element (wt.%)	
	Carbon	Hydrogen
SiO_2_	0.20	0.21
MVF-SiO_2_-1	3.71	0.79
MVF-SiO_2_-2	4.17	0.82
MVF-SiO_2_-4	7.48	1.29
MVF-SiO_2_-16	16.04	2.31

**Table 6:** Mechanical properties of silicone rubber/silica filler composites.

Sample	Tensile Strength (MPa)	Elongation at Break (%)	Hardness (Shore A)
Silicone rubber/ SiO_2_	5.99	232.21	65
Silicone rubber/MVF-SiO_2_-1	3.28	138.58	64
Silicone rubber/MVF-SiO_2_-2	2.90	124.30	63
Silicone rubber/MVF-SiO_2_-4	2.86	123.60	62
Silicone rubber/MVF-SiO_2_-16	1.64	73.02	55

**Table 7:** Silicon concentration of the residual solutions obtained from the silicone rubber/silica filler composites, as determined by ICP-OES analysis.

	Silicon Concentration (mg/L)
Silicone rubber/SiO_2_	65.5 ± 3.1
Silicone rubber/MVF-SiO_2_-1	5.9 ± 0.4
Silicone rubber/MVF-SiO_2_-2	5.5 ± 0.3
Silicone rubber/MVF-SiO_2_-4	4.8 ± 0.6
Silicone rubber/MVF-SiO_2_-16	4.6 ± 0.7

## Data Availability

Data are contained within the article.
